# From general base to general acid catalysis in a sodium-specific DNAzyme by a guanine-to-adenine mutation

**DOI:** 10.1093/nar/gkz578

**Published:** 2019-07-05

**Authors:** Lingzi Ma, Sanjana Kartik, Biwu Liu, Juewen Liu

**Affiliations:** Department of Chemistry, Waterloo Institute for Nanotechnology, University of Waterloo, Waterloo, Ontario N2L 3G1, Canada

## Abstract

Recently, a few Na^+^-specific RNA-cleaving DNAzymes were reported, where nucleobases are likely to play critical roles in catalysis. The NaA43 and NaH1 DNAzymes share the same 16-nt Na^+^-binding motif, but differ in one or two nucleotides in a small catalytic loop. Nevertheless, they display an opposite pH-dependency, implicating distinct catalytic mechanisms. In this work, rational mutation studies locate a catalytic adenine residue, A22, in NaH1, while previous studies found a guanine (G23) to be important for the catalysis of NaA43. Mutation with p*K*_a_-perturbed analogs, such as 2-aminopurine (∼3.8), 2,6-diaminopurine (∼5.6) and hypoxanthine (∼8.7) affected the overall reaction rate. Therefore, we propose that the N1 position of G23 (p*K*_a_ ∼6.6) in NaA43 functions as a general base, while that of A22 (p*K*_a_ ∼6.3) in NaH1 as a general acid. Further experiments with base analogs and a phosphorothioate-modified substrate suggest that the exocyclic amine in A22 and both of the non-bridging oxygens at the scissile phosphate are important for catalysis for NaH1. This is an interesting example where single point mutations can change the mechanism of cleavage from general base to general acid, and it can also explain this Na^+^-dependent DNAzyme scaffold being sensitive to a broad range of metal ions and molecules.

## INTRODUCTION

DNAzymes are DNA sequences with catalytic activities (1–3). A variety of RNA-cleaving DNAzymes require various monovalent (e.g. Na^+^ (4,5), Ag^+^ (6)), divalent (e.g., Pb^2+^ (1), Zn^2+^ (7), Cu^2+^ (8), UO_2_^2+^ (9), Hg^2+^ (10), Cd^2+^ (11)) and trivalent (e.g. lanthanides (12)) metal ions for catalysis. DNAzymes are highly attractive for their excellent stability, programmability and cost-effectiveness, leading to interesting applications in biosensing and therapeutics (13–16). However, our understanding of DNAzyme catalysis is still limited compared to the substantial knowledge in ribozyme catalysis due to their rich structural biology data. Most self-cleaving ribozymes (e.g. the Rzb hammerhead (17,18), *env25* pistol (19,20) and twister ribozymes (21)) use a general base mechanism, where a guanine helps activate the 2′-OH nucleophile. Adenines or cytosines can play the general acid role in the pistol (19), twister (21) and hepatitis delta virus (HDV) ribozymes (22).

The involvement of polyvalent metal ions may complicate data analysis. For mechanistic studies, monovalent-metal-dependent enzymes are more attractive. The first Na^+^-dependent DNAzyme, NaA43 was reported in 2015 by Lu *et al.* (4). Interestingly, its conserved sequence is quite similar to a lanthanide-dependent DNAzyme named Ce13d (23–25). Their overlapping sequence was revealed to be part of a Na^+^-binding aptamer, which is responsible for their Na^+^ specificity (25,26). Extensive studies have been performed on the aptamer part to understand specific Na^+^ binding (27,28).

Recently, we reported the NaH1 DNAzyme, which differs from the NaA43T DNAzyme (a mutated shorter version of NaA43) by only two nucleotides in the enzyme loop (29). An important guanine in NaA43 was replaced by an adenine in NaH1, and NaH1 also requires Na^+^ for activity. An interesting difference between them is their pH-dependency: the optimal pH being 7 for NaA43 and 6 for NaH1. Such DNAzymes are ideal for mechanistic studies since no polyvalent metals are involved, and catalysis is likely achieved via nucleobases. In this work, we performed careful biochemical and spectroscopic experiments, providing compelling evidence that on the same DNAzyme scaffold, a simple point mutation can switch the mechanism from being general base (for NaA43) to general acid (for NaH1) catalysis. The tolerance of different mechanisms may also explain the activity of this scaffold (e.g. Ce13d) with a diverse range of metal ions and even non-metals.

## MATERIALS AND METHODS

### Chemicals

All the DNA samples used in this work were purchased from Integrated DNA Technologies (Coralville, IA). Sodium chloride, lithium chloride, potassium chloride, acetate acid, 2-(*N*-morpholino)ethanesulfonic acid (MES), 2-[4-(2-hydroxyethyl)piperazin-1-yl]ethanesulfonic acid (HEPES), and 3-(*N*-morpholino)propanesulfonic acid (MOPES) were from Mandel Scientific (Guelph, ON, Canada). Lithium hydroxide was purchased from Alfa Aesar. All of the solutions and buffers used in this work were prepared with Milli-Q water. All the DNA sequences used in this work are listed in Supplementary Table S1.

### Activity assays

The FAM-labeled substrate (2 μM) was respectively hybridized with each enzyme or their mutants (3 μM) in the reaction buffer to form the DNAzyme complexes. The mixtures were annealed by heating at 93°C for 1 min and then gradually cooling down to 4°C for over 30 min. The DNAzyme complexes were then diluted in reaction buffer (6 μl) to 0.3 μM. A small volume (1 μl) of NaCl was then added to initiate the cleavage reaction with a final concentration of 10 mM Na^+^. At each time point, a 7 μl reaction solution was quenched with 8 μl of 8 M urea. The cleavage products were quantified by 15% denaturing polyacrylamine gel electrophoresis (dPAGE) and analyzed by a Bio-Rad Chemi-Doc MP imaging system. Reaction buffer A (50 mM MES, pH 6.0, 25 mM LiCl) and buffer B (50 mM MOPES, pH 7.0, 25 mM LiCl) were used.

Kinetic data were fit with the first-order equation, ${\rm{\% \ }}{P_{cleavage,t}} = \ \% {P_{max}}( {1 - {e^{ - {k_{obs}}t}}} )$, where %*P*_max_ is the maximum cleavage yield at the end of the reaction and ${k_{obs}}$ is the cleavage rate constant. For pH-rate profiles, acetate buffer (50 mM) was used for pH 4.0 and 4.5; MES buffer (50 mM) for pH 5.5–6.5; and MOPES buffer (50 mM) for pH 7.0–8.0. All the buffer solutions were adjusted by 1 M LiOH to eliminate background Na^+^. The pH-rate profiles of NaH1 and NaA43 were fit with equations ${k_{obs}} = \frac{{{k_{max}}}}{{1 + {{10}^{( {pH - p{K_a}} )}}}}$ and ${k_{obs}} = \frac{{{k_{max}}}}{{1 + {{10}^{( {p{K_a} - pH} )}}}}$ respectively, where ${k_{obs}}$ is the observed rate constant, and ${k_{max}}$ is the maximal observed rate constant.

### Phosphorothioate substitution

The FAM-labeled PS-modified substrates, *R*_p_ and *S*_p_, were separated using HPLC as described previously (11). The substrates were annealed with the DNAzymes with a ratio of 1:1.5. Cleavage kinetics was measured in the presence of 10 mM NaCl for 1 h in reaction buffer A for NaH1 or buffer B for NaA43T. In the metal rescue assays, CdCl_2_ was introduced in the reaction with a final concentration of 2 mM.

### Fluorescence spectroscopy

The DNAzyme complex was prepared by annealing the 2-aminopurine (2AP)-modified enzyme and non-modified substrate with a ratio of 1:2 in buffer A (50 mM MES, 25 mM LiCl, pH 6.0). The mixture was heated to 93°C for 1 min followed by gradual cooling down to 4°C. With excitation at 310 nm, the emission was measured from 360 to 450 nm. Small volumes of chloride salts were gradually titrated into the sample to achieve a final concentration up to 45 mM. After a quick mix, the fluorescence signal was immediately measured in a 1 × 1 cm quartz fluorescence cuvette using a Cary Eclipse fluorometer. The emission intensity enhancement at 370 nm was normalized by *F*/*F*_0_, where *F*_0_ and *F* represent the fluorescence signal before and after the addition of Na^+^, respectively. The *K*_d_ value was obtained by fitting into the one-site binding equation:$F = {F_0}\ + a \cdot [ {{M^ + }] {/({k_d} + } [{M^ + }} ])$. [M^+^] is the metal concentration, and $a$ is the fluorescence change when [M^+^] = ∞.

## RESULTS AND DISCUSSION

### The NaA43 and NaH1 DNAzymes

**Figure 1.**
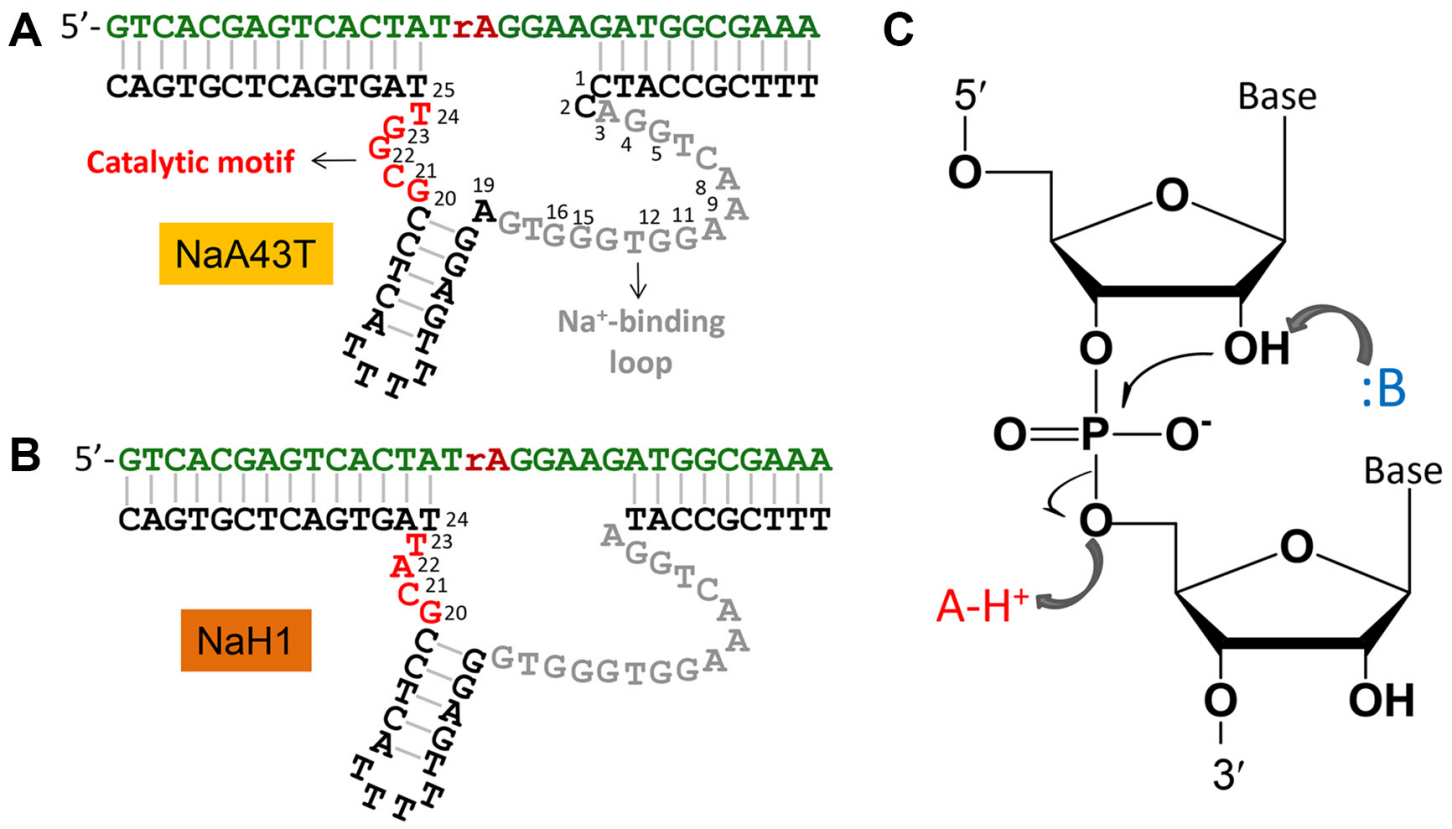
The secondary structures of two Na^+^-specific RNA-cleaving DNAzymes named (**A**) NaA43T and (**B**) NaH1. The key catalytic motifs are the small loops in red. (**C**) A general RNA cleavage reaction requires the nucleophilic attack from 2′-OH resulting in the leaving of the 5′-OH group, which can be accelerated by the general acid/base catalysis.

The secondary structures of the NaA43T and NaH1 DNAzymes are shown in Figure 1A and B, respectively. NaA43T is a truncated version of NaA43 with retained specificity for Na^+^ (25). These DNAzymes have the same substrate that contained a single RNA linkage (rA for ribo-adenine) serving as the cleavage site. NaA43T and NaH1 have the same 16-nt Na^+^-binding motif. The catalytically important regions are drawn in red. Our previous study indicated that the G23 residue in NaA43 is highly conserved (25), but no guanine is present in the corresponding region of NaH1. Therefore, these two DNAzymes might have distinct catalytic mechanisms.

The catalysis of many small self-cleaving ribozymes has been thoroughly studied (30–32). In general, the RNA cleavage reaction is initiated by a nucleophilic attack from the 2′-OH of the ribose to the scissile phosphate, resulting in the leaving of the 5′-oxygen of the next nucleotide (Figure 1C). In self-cleaving ribozymes, the cleavage reaction is often accelerated by a general base (e.g., a guanine or a metal bound water after deprotonation) (19,33) to help deprotonate the 2′-OH, or by a general acid (e.g. adenine or cytosine) (22,34) to neutralize the developing negative charge on the 5′-oxygen (32). This study intends to compare these two Na^+^-specific DNAzymes for mechanistic insights taking advantage that no polyvalent metal ions are involved.

### Rational evolution from NaA43T to NaH1

**Figure 2.**
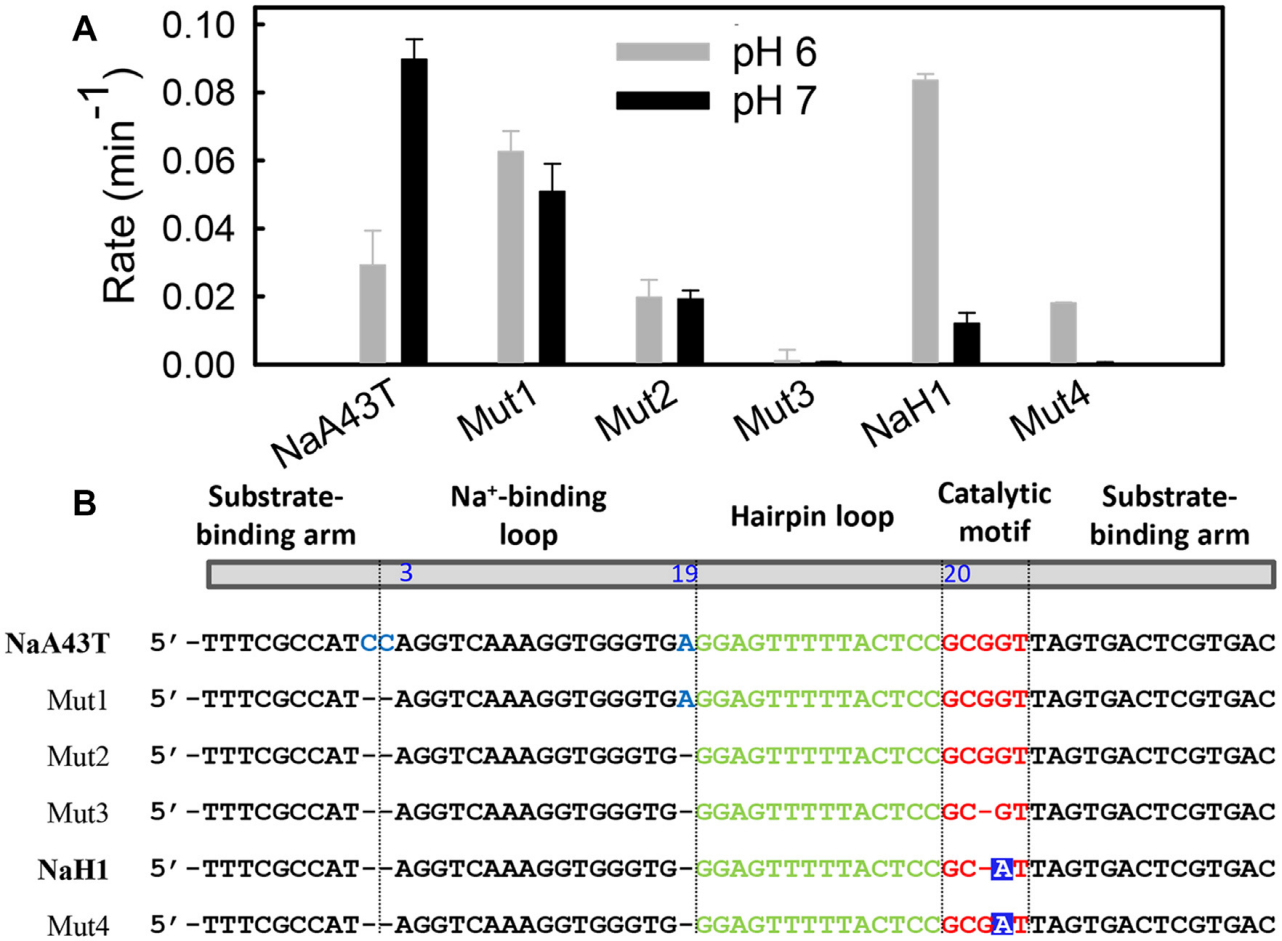
(**A**) The cleavage rate of each mutant in the presence of 10 mM NaCl at pH 6 (gray bars) and pH 7 (black bars). (**B**) List of the DNAzyme sequences used in this study. The A22 residue is highlighted with blue squares.

Since these two DNAzymes are highly similar, we gradually mutated one DNAzyme into the other to identify important residues. As shown in Figure 2A, the cleavage rate of NaA43T measured at pH 7 was ∼3-fold faster than that at pH 6. Before modifying the catalytic motif, we first deleted the C1 and C2, and then A19 in NaA43T (Mut1 and Mut2 in Figure 2B). Mut1 and Mut2 showed gradually decreasing activity, but their activity became similar at both pH’s. Based on Mut2, we further deleted G22 (or G23) in the small loop resulting in Mut3, which had almost no activity detected at either pH values. Based on our previous study, NaA43 was still active when G22 was mutated to A, T or C (25). However, deleting G22 inhibited the cleavage activity in this study. Therefore, G22 is likely to play a structural role (e.g. a spacer) to position the catalytically important G23 for catalysis.

Based on Mut3, we mutated G23 into an adenine and NaH1 was obtained (this adenine is A22 in NaH1). NaH1 exhibited a higher cleavage rate under pH 6, while its activity at pH 7 remained low. We also added a guanine back to position 22 (Mut4), whose activity decreased at both pH’s, but the pH 6 activity was still higher than that at pH 7. Therefore, the activity of Mut4 is more similar to NaH1 than to NaA43T. Overall, the highest activity was at pH 7 when G23 was present, and was at pH 6 when A22 was present. Based on this experiment, we can define an enzyme to be from NaA43T if it has G23 and the activity at pH 7 is higher or comparable to that at pH 6. On the other hand, if the enzyme has A22 and its activity is higher at pH 6, it is NaH1. If these nucleotides are directly involved in catalysis, they likely have very different reaction mechanisms, since G and A can play different acid/base catalysis roles (31,32).

### The A22 in NaH1 is highly conserved

**Figure 3.**
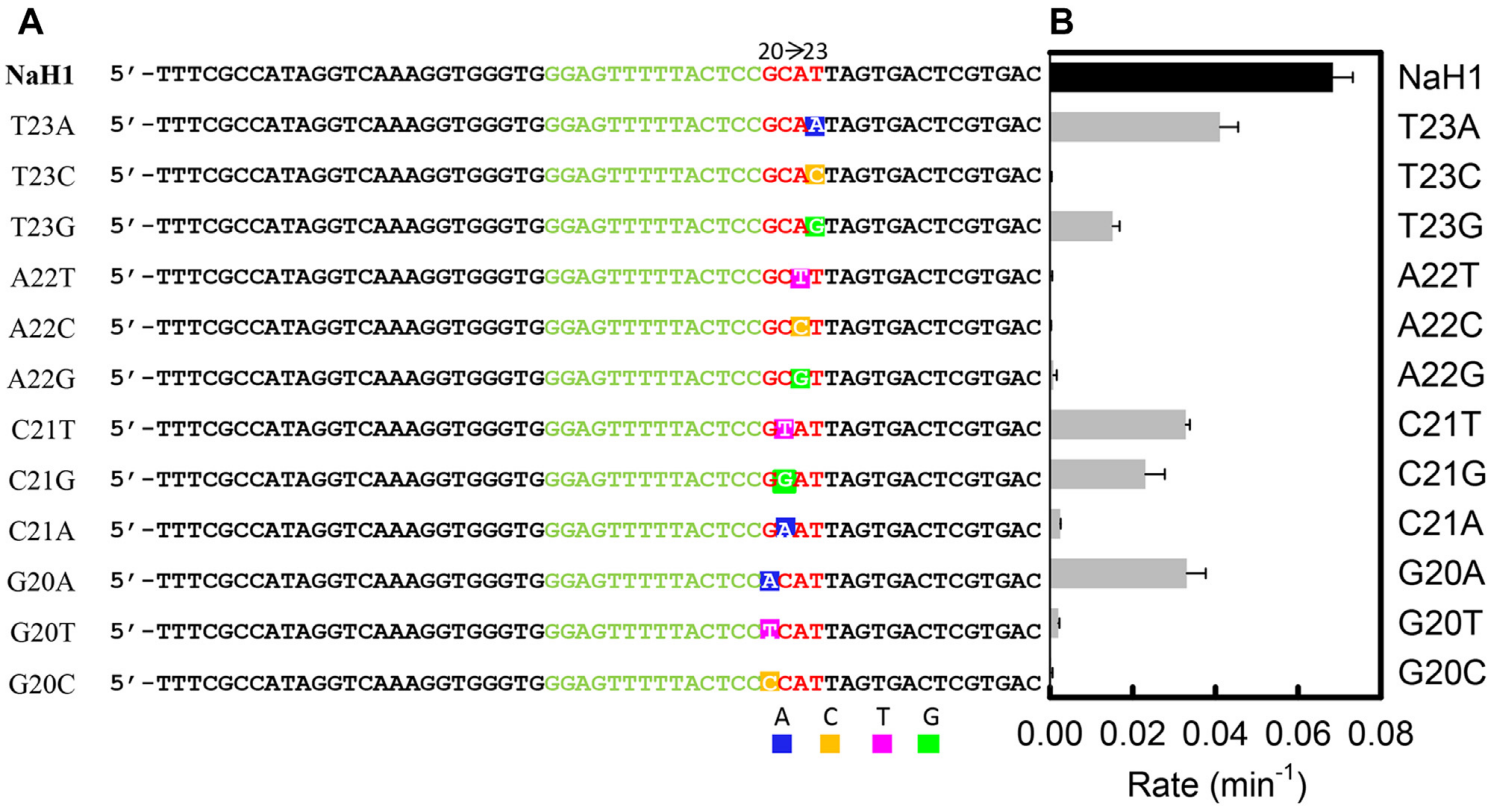
(**A**) The sequences used in this point mutaion study based on NaH1 from G20 to T23. (**B**) The cleavage rate of each mutant measured with 10 mM NaCl in pH 6 buffer.

To further locate important nucleotides in the catalytic loop of NaH1, point mutaions were performed (Figure 3A). The cleavage rate of each mutant was measured at pH 6 (Figure 3B). Mutating T23 to A resulted in a ∼1.6-fold reduction in rate, while mutating C21 to T or G decreased the rate by 2 to 3-fold. Besides, purine-to-purine replacement (G20A) was tolerated with ∼50% rate remaining, which was also observed for the G20 site in NaA43T (25). On the contrary, mutation of A22 to T, C or G led to a ∼100–1000-fold decrease in rate. Therefore, A22 plays a crucial role in the cleavage reaction, which is in agreement with the above rational evolution experiments.

### pH-rate profiles

**Figure 4.**
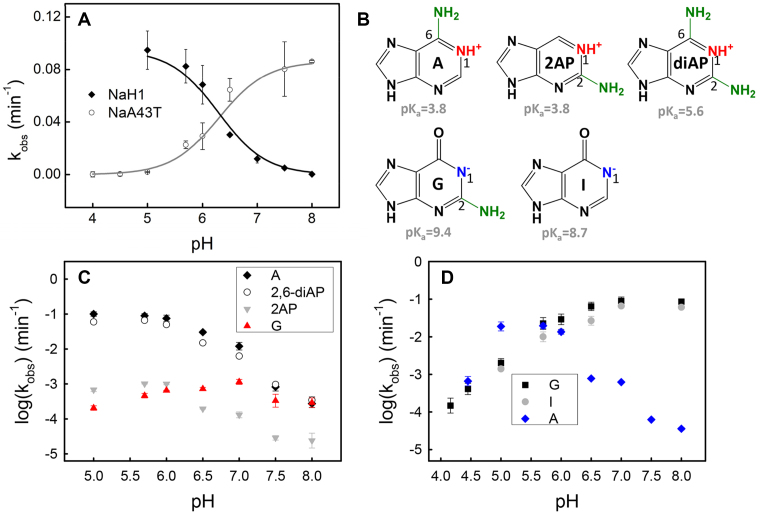
(**A**) pH-rate profiles of NaH1 and NaA43T in the presence of 10 mM Na^+^. The p*K*_a_ of the general acid catalyst in NaH1 was 6.3 ± 0.4 (*R*^2^ = 0.92), while the p*K*_a_ of the general base in NaA43T was 6.6 ± 0.1 (*R*^2^ = 0.97). (**B**) Chemical structures and p*K*_a_ values of adenine, 2-aminopurine (2AP), 2,6-diaminopurine (2,6-diAP), guanine, and hypoxanthine (I). (**C**) pH-rate profiles of the wild-type NaH1 and its 2,6-diAP-, 2AP- and G-substitutions in the presence of 10 mM NaCl. (**D**) pH-rate profiles of the wild-type NaA43T and its I- and A-substitutions in the presence of 10 mM NaCl.

Catalytically important guanines often play a general base role in ribozymes such as the Rzb hammerhead (17,18), *env25* pistol (19,20), and twister ribozymes (21). The p*K*_a_ of the N1 position of guanine is ∼9.4, which can be lowered toward neutrality by coordinating with nearby nucleobases or metal ions, allowing its general base role at neutral pH (35,36). Adenine, on the other hand, is often known for its general acid role in stabilizing the leaving group (i.e. 5′-*O*) (19,21,34). To better understand the reaction mechanism, we carefully examined the pH-rate profiles. For NaA43T, we observed a gradual increase in cleavage rate with increasing pH, plateauing at pH 7.5 (Figure 4A, circles). Such a pH-rate profile implies that a single general base functions during the cleavage reaction until it is fully deprotonated. In contrast, a declining trend was observed for NaH1 between pH 5.0 to 8.0 (Figure 4A, diamonds), indicating a general acid catalysis and its protonation was inhibited by raising pH.

In the mutation studies, G23 and A22 were determined to be crucial for the activity of NaA43 and NaH1, respectively. Taken together, we propose that G23 functions as a general base in NaA43T, while A22 as a general acid in NaH1 (see base analog studies below for further evideince). The p*K*_a_ of the general base in NaA43T shifts from ∼9.4 (N1 position) to 6.6 ± 0.1 (Figure 4A, gray curve). For NaH1, a shifted p*K*_a_ of 6.3 ± 0.4 (Figure 4A, black curve) for the catalytic adenine was observed in the presence of 10 mM Na^+^.

It is very interesting that on the same DNAzyme scaffold, by just mutating one or two nucleotides, the catalytic mechanism is completely changed. Considering the very small size of these DNAzymes, the tolerance of different catalytic mechanisms by point mutations has not been seen previously. The closest example is a hamerhead ribozyme, where mutating an invariant guanine to adenine retained its general base role for the catalysis (instead of general acid) (17). Meanwhile, this mutant exhibited a 13 000-fold lower rate and required a high Mg^2+^ concentration.

This tolerating property and switchable general acid/base catalysis mechanism have reminded us of another related DNAzyme called Ce13d, which requires both Ce^3+^ (or another trivalent lanthanide) and Na^+^ for activity. Ce13d has the same Na^+^ binding motif, but the small catalyitc loop is almost completely eliminated (Ce^3+^ to carry out the catalytic role). The Ce13d DNAzyme was extremely tolerant for various metal ions, including all the trivalent lanthanides, Y^3+^ (23), a low activity with Cr^3+^ (37), and a moderate activity with Pb^2+^ (23). After making a single phosphorothioate modification at the cleavage site, it became active with all thiophilic metals such as Hg^2+^, Cd^2+^, Pb^2+^, Tl^3+^ and Au^3+^ (38). It can even accelerate the cleavage of the PS substrate by I_2_, a non-metal (39). The activity with various metal ions and even elimination of polyvalent metal ions indicates that the DNAzyme scaffold is highly tolerant for catalysis. Thus, the swithcing of general acid/base mechanism might also be quite reasonable.

### Base analogs further probing catalytic mechanism

To confirm the general acid role of A22 in NaH1, we tested its mutants including using base analogs. A22 has three possible positions to donate protons to the 5′-*O*: N1, N3 and N7. In small ribozymes, the N1 and N3 positions were reported for general acid catalysis (19,21,40). With a higher p*K*_a_ (41), N1 is more likely to donate protons. In this work, a series of p*K*_a_-perturbed analogs (Figure 4B), such as 2-aminopurine (∼3.8), 2,6-diaminopurine (∼5.6), and guanine (∼9.4) were tested (Figure 4C). The pH-rate profile of the 2,6-diaminopurine substitution (2,6-diAP, circles) overlapped with that of adenosine (diamonds). Mutating A to 2-aminopurine (2AP, grey triangles) decreased the activity by ∼2 orders of magnitude, but its overall profile was parallel to that of the wild type NaH1. Therefore, the 6-amino group of A22 is important for catalytic rate, likely by forming hydrogen bonding to stabilize the transition state (31). With their N1 positions still available for protonation, these analogs displayed a similar pH-dependent activity plateauing at pH <6. When substituting A with G (red triangles), a gradual rate decrease was observed below pH 7, with its maximum activity occurring at a higher pH. Therefore, the N1 position of A22 is indeed responsible for the general acid catalysis of NaH1.

In NaA43T, substitution at G23 with hypoxanthine (I, p*K*_a_ ∼ 8.7) and adenine (A, p*K*_a_ ∼ 3.8) was tested to determine its catalytic role (Figure 4D). The G to I mutation (gray circles) did not affect its pH-rate dependence but gave a lower rate between pH 5.0 and 7.0. The 2′-exocyclic amino group of G23 could better stabilize the transition state likely by hydrogen bonding (42). On the contrary, mutating G23 into adenine resulted in a bell-shaped pH-rate profile with the highest activity at pH ∼5.5, similar to that of NaH1 (blue diamonds). Therefore, the N1 position of G23 is likely to directly participate in the catalysis by serving as a general base.

**Figure 5.**
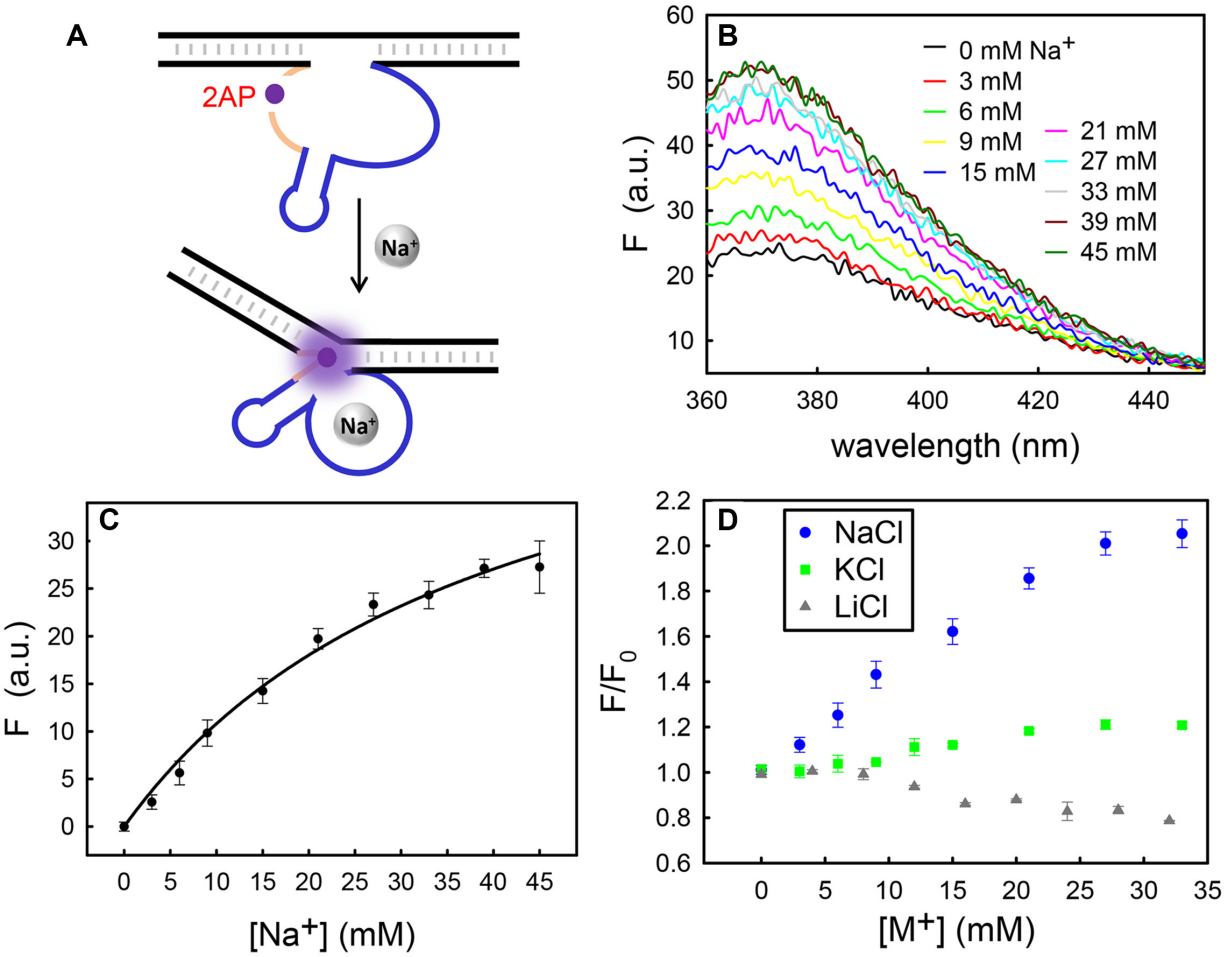
(**A**) Using 2AP to probe the local environment of the catalytic adenine due to Na^+^ binding. (**B**) The fluorescence spectrum of the A22-to-2AP mutant after titrating NaCl. (**C**) The 2AP fluorescence at 370 nm versus the Na^+^ concentration. (**D**) Fluorescence titration with Na^+^, K^+^ or Li^+^.

The above mutation studies primarily focused on general acid/base catalysis. After mutation, the loss of activity could also be due to perturbed enzyme folding or Na^+^ binding. The fluorescence of 2AP is highly sensitive to its nearby base stacking environment (43). Thus, 2AP has been frequently used to probe local folding of DNAzymes and ribozymes (44–46). We then substituted A22 by 2AP (Figure 5A). In activity assays, the enzyme bearing the A22–2AP mutation retained its cleavage activity with a ∼100-fold lower rate compared to the wild type (Figure 4C, gray triangles). However, titrating NaCl induced a gradual increase in its fluorescence (Figure 5B). The fluorescence at 370 nm was further plotted as a function of Na^+^ concentration showing binding curve with a *K*_d_ of ∼40 mM Na^+^ (Figure 5C). To confirm the Na^+^ specificity, Li^+^ and K^+^ were also titrated. The intensity barely changed (<20%) with up to ∼30 mM KCl, while a slight fluorescence decrease was observed with LiCl (Figure 5D). Therefore, the 2AP-substitution does not affect the enzyme folding upon Na^+^ binding. Therefore, the activity drop of the A22-to-2AP mutant was likely caused by destabilizing the hydrogen bonding on the 6-amino of A22 instead of perturbed sodium binding. An enhanced fluorescence suggests a weakened base-base stacking near the catalytic residue (43), supporting the importance of A22 to the catalysis. A22 is likely to be released from stacking interactions from the neighbor bases due to Na^+^ binding, and exposed to the active site. As shown in Figure 4C, the pH-rate profile of NaH1 revealed a slope close to 1 in the high pH region, indicating one proton transfer is the rate-limiting step. A similar relationship was also seen in the low pH region of NaA43T. This supports the idea that chemistry is the rate-limiting step instead of the folding.

### Phosphorothioate substitution and metal rescue

**Figure 6.**
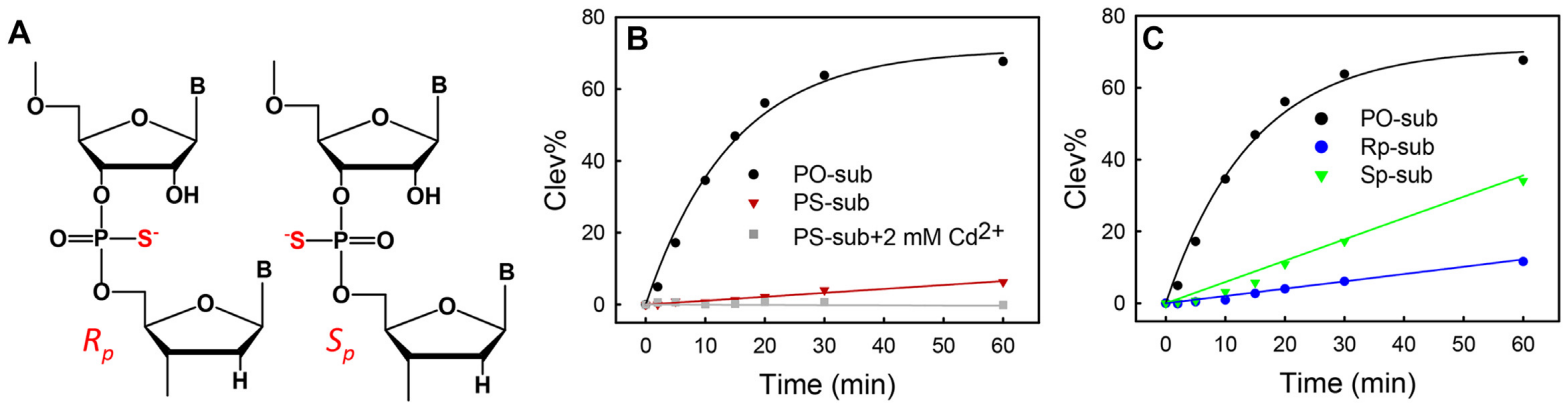
(**A**) The *R*_p_ and *S*_p_ phosphorothioate diastereomers at the cleavage junction. (**B**) The comparison between the normal PO-substrate and the PS-substrates of NaH1 in the presence of 10 mM NaCl with or without 2 mM Cd^2+^. (**C**) Cleavage activity of NaH1 using PO-, *R*_p_- or *S*_p_-substrates in the presence of 10 mM NaCl.

In self-cleaving ribozymes, non-bridging oxygens at the scissile phosphate often play important roles in directly coordinating with catalytic metal ions or nucleobases. Phosphorothioate (PS) substitution has been widely applied in probing the importance of non-bridging oxygens (24,47). Previous biochemical studies indicate that the Na^+^ binding site in NaA43 is the 16-nt loop (Figure 1A), instead of the cleavage site (25). To test metal binding at the cleavage site, the activity of NaH1 with the PS-substrates was examined in the presence of 10 mM Na^+^. First, a racemic mixture of the PS substrate was used, which contained an equal amount of the *R*_p_ and *S*_p_ diastereomers as shown in Figure 6A.

The cleavage rate with the PS-substrate was ∼68-fold slower than the PO-substrate (Figure 6B), showing a normal thio effect at 10 mM Na^+^. A careful examination of the kinetic data can reveal that the cleavage did not have a fast phase and the yield was very low. Normally, for metal-based interactions, if the metal binds specifically to one of the non-bridging oxygens, one of the stereoisomers should remain active (48). However, it is not the case here. In addition, adding thiophilic Cd^2+^ could not rescue the activity, also disapproving metal/phosphate interactions. For comparison, the lanthanide-dependent Ce13d DNAzyme has a normal thio effect which can be fully rescued by Cd^2+^ (24). Therefore, the role of Na^+^ is to bind to the aptamer loop instead of directly interacting with the scissile phosphate.

The loss of activity with PS-substrates could be due to a diminished hydrogen bonding between catalytic A22 and non-bridging oxygens caused by the sulfur substitution. The importance of the exocyclic amine of A22 has been addressed in the activity study of A22-to-2AP mutant. We further measured the activity of NaH1 with the two diastereomers *R*_p_ and *S*_p_ (Figure 6A). The cleavage rates of both *R*_p_ and *S*_p_ substrates were slower than the PO-substrate (Figure 6C). With a relatively lower activity, the *pro*-*R*_p_ oxygen seems to contribute more to interacting with the catalytic adenine at the cleavage site. Notably, the *pro*-*R*_p_ oxygen was reported to be more important in the catalysis of many RNA-cleaving ribozymes and DNAzymes including the hammerhead (49), the hepatitis delta virus (HDV) ribozymes (47,48), and the Ce13d DNAzymes (24). For example, in the HDV ribozyme, the *pro*-*R*_p_ oxygen directly interacts with a Mg^2+^ ion and is within hydrogen bonding distance of a catalytic C75 according to its crystal structure (48,50).

In addition, the activity difference between the *R*_p_- and *S*_p_-substitutions was only ∼3-fold, suggesting that both of the non-bridging oxygens are involved in interacting with the catalytic adenine. A similar difference was also seen in a study of the activity of Ce13d (<10-fold) with the PS-substrates (24). As a comparison, the activity of the *S*_p_ was >100-fold faster than the *R*_p_ in the hammerhead ribozyme (49,51). The importance of the 6-amino group in A22 has been addressed in the previous mutation study. Together, we propose that both of the non-bridging oxygens can form hydrogen bonding with the 6-amino of A22 in NaH1. The *pro*-*R*_p_ oxygen likely plays a slightly more important role than the *pro*-*S*_p_ oxygen. The involvement of both oxygens might also contribute to the tolerance to different catalytic mechanisms.

**Figure 7.**
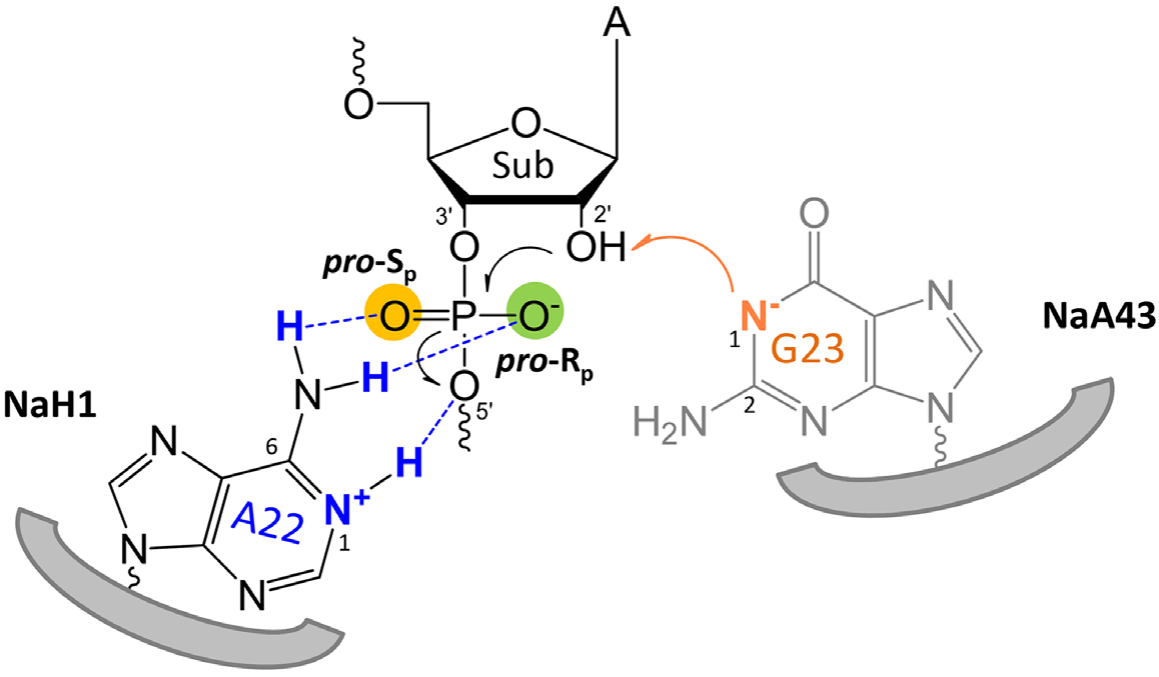
A model describing the general acid/base mechanism of the NaH1 and NaA43 DNAzymes in the RNA cleavage reaction.

Finally, we have analyzed the cleavage product of NaH1 using mass spectrometry (Supplementary Figure S1). The 5′-fragment of the cleaved substrate terminated with a 2′,3′-cyclic phasphate. Therefore, a normal RNA cleavage mechanism was confirmed. Taken together, Figure 7 schematically illustrates the catalytic mechanism of the Na^+^-dependent RNA-cleaving DNAzymes from this study. In NaH1, a N1-protonated adenine (A22) with a shifted p*K*_a_ of ∼6.3 serves as a general acid, which could stabilize the negative charge on the 5′-leaving oxygen. Meanwhile, 2AP- and PS-substitution assays suggested that 6′-exocyclic amine directly participates in the cleavage by forming the hydrogen bonding with both of the non-bridging oxygens. In the wild-type NaA43, the N1 position of G23 functions as a general base with its p*K*_a_ shifted to ∼6.6 during the reaction. Its deprotonated form facilitates the deprotonation of the 2′-OH, which then performs the nucleophilic attack on the scissile phosphate. Due to their high sequence similarity, we propose that the Na^+^-dependent DNAzyme folds into a defined structure upon Na^+^ binding with catalytic purine bases at the active site accelerating the RNA cleavage.

## CONCLUSIONS

The NaA43 and NaH1 DNAzymes were selected separately under different selection conditions. The pH optima of each DNAzyme matched the pH of their respective selection buffers. Despite their high sequence similarity, the minor differences near the cleavage site resulted in distinct pH preferences. In this work, rational evolution studies revealed a 4 to 5-nt catalytic motif in these DNAzymes. Furthermore, a series of mutation studies in NaH1 demonstrated that A22 is indispensable for its catalysis, and previous studies indicated that G23 plays an important role in NaA43. Combining with pH-rate profiles of their base analogs, the N1 position of A22 (p*K*_a_ shifted to ∼6.3) functions as a general acid in NaH1, while N1 of G23 (p*K*_a_ shifted to ∼6.6) serves as a general base in NaA43. Base analogs revealed the importance of 6-amine in A22 for the catalysis. To our knowledge, this is the first report of a shift between general acid and base catalysis mechanism by mutating only one or two nucleotides in a conserved DNAzyme scaffold. Notably, this scaffold is also active with a diverse range of polyvalent metal ions by removing the current catalytic motif. Such tolerance may explain the ability to use distinct reaction mechanisms on such a small structure. Future studies may include NMR spectroscopy to directly monitor the actual p*K*_a_ values of critical residues in the DNAzymes and study kinetic isotope effects for probing proton transfer.

## Supplementary Material

gkz578_Supplemental_File
