# Supplementary material for: From general base to general acid catalysis in a sodium-specific DNAzyme by a guanine-to-adenine mutation
Source: Nucleic Acids Res. 2019 Jul 5;47(15):8154–62. doi: 10.1093/nar/gkz578 (PMC6736077; doi:10.1093/nar/gkz578)
Supplement: gkz578_Supplemental_File [file gkz578_supplemental_file.pdf]

Supplementary Data

From general base to general acid catalysis in a sodium-specific DNAzyme by a guanine-to-adenine mutation

Lingzi Ma, Sanjana Kartik, Biwu Liu, and Juewen Liu*

Department of Chemistry, Waterloo Institute for Nanotechnology, University of Waterloo, Waterloo, Ontario, N2L 3G1, Canada

Email: liujw@uwaterloo.ca

Table S1. DNA sequences used in this work. FAM = carboxyfluorescein; rA = adenine ribonucleotide; /2AP/ = 2-aminopurine; /2,6-diAP/ = 2,6-diaminopurine; /I/ = hypoxanthine. The asterisk denotes for a PS modification. The individual mutants are not listed here and they can be obtained based on Figure 2B and Figure 3A.

**DNA Names**	**Sequences and modifications**
FAM-PO-Sub	GTCACGAGTCACTAT**rA**GGAAGATGGCGAAA-**FAM**
NaH1	TTTCGCCATAGGTCAAAGGTGGGTGGGAGTTTTACTCCGCATTAGTGA CTCGTGAC
NaA43T	TTTCGCCATCCAGGTCAAAGGTGGGTGAGGAGTTTTACTCCGCGGTTA GTGACTCGTGAC
NaH1-G	TTTCGCCATAGGTCAAAGGTGGGTGGGAGTTTTACTCCGCGTTAGTGA CTCGTGAC
NaH1-2AP	TTTCGCCATAGGTCAAAGGTGGGTGGGAGTTTTACTCCGC/**2AP**/TTAGT GACTCGTGAC
NaH1-2,6diAP	TTTCGCCATAGGTCAAAGGTGGGTGGGAGTTTTACTCCGC/**2,6-diAP**/TTAGTGACTCGTGAC
NaA43T-A	TTTCGCCATCCAGGTCAAAGGTGGGTGAGGAGTTTTACTCCGCGATTA GTGACTCGTGAC
NaA43T-I	TTTCGCCATCCAGGTCAAAGGTGGGTGAGGAGTTTTACTCCGCG/**I**/TTA GTGACTCGTGAC
Sub	GTCACGAGTCACTAT**rA**GGAAGATGGCGAAA
Sub-dA	GTCACGAGTCACTATAGGAAGATGGCGAAA
FAM-PS-Sub	GTCACGAGTCACTAT**rA***GGAAGATGGCGAAA-FAM

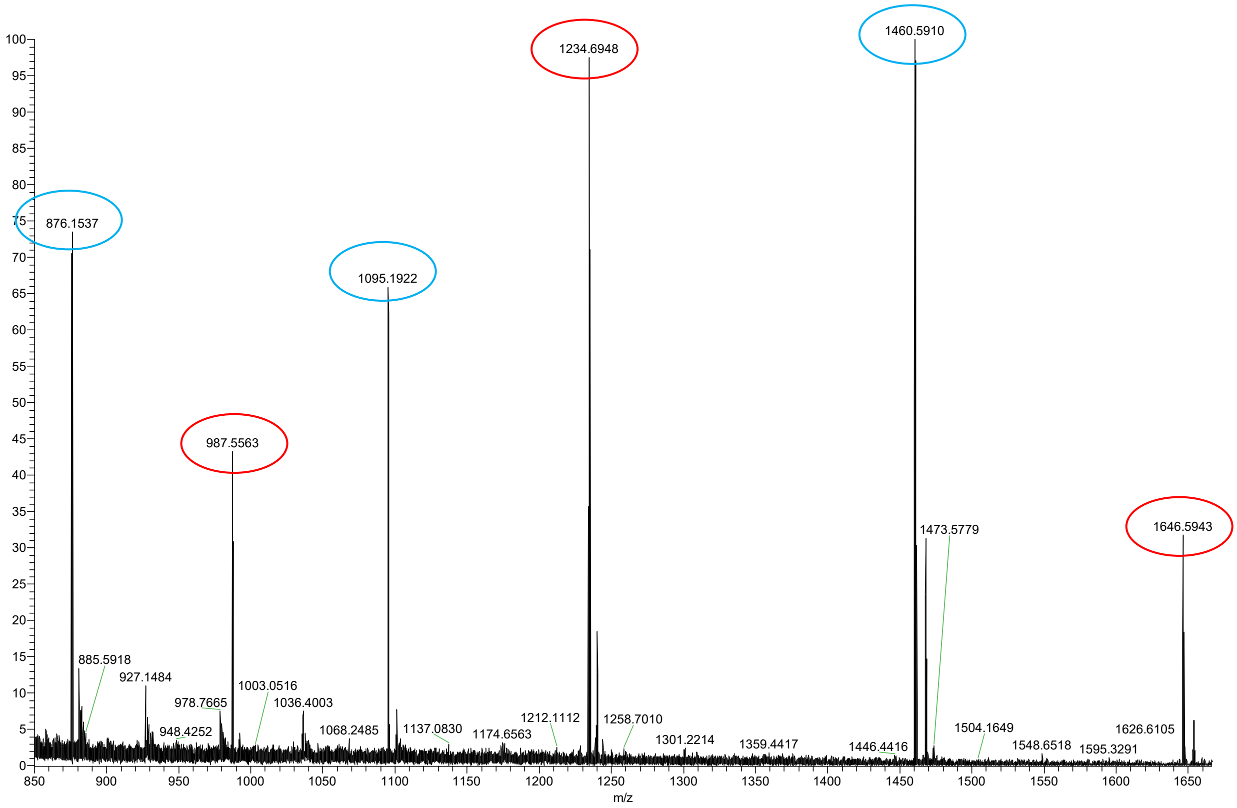

Figure S1. Mass spectrometry characterization of the NaH1 cleavage product. The red peaks represent the 5′-fragment of the cleaved-substrate containing a 2′, 3′-cyclic phosphate group (MW=4942.7 Da, 3, 4 and 5 charges for three marked peaks). The blue peaks represent the 3′-fragment of the cleaved-substrate (MW=4384.7 Da. 3, 4 and 5 charges for the three marked peaks). The sample was prepared by reacting non-labeled substrates (**Table S1**) with NaH1 DNAzymes (in 1:1.5 ratio) in 50 mM MES buffer (pH 6, 25 mM LiCl, 100 mM NaCl) for overnight. A Sep-Pak column was further used to desalt the sample. After dried, the sample was re-suspended in 20 μL Milli-Q water with a final substrate concentration of ~50 μM and analyzed with an ESI mass spectrometer.
